# Exposure to Bisphenol A Analogs and the Thyroid Function and Volume in Women of Reproductive Age—Cross-Sectional Study

**DOI:** 10.3389/fendo.2020.587252

**Published:** 2021-01-19

**Authors:** Justyna Milczarek-Banach, Dominik Rachoń, Tomasz Bednarczuk, Katarzyna Myśliwiec-Czajka, Andrzej Wasik, Piotr Miśkiewicz

**Affiliations:** ^1^Department of Internal Medicine and Endocrinology, Medical University of Warsaw, Warsaw, Poland; ^2^Department of Clinical and Experimental Endocrinology, Medical University of Gdańsk, Gdańsk, Poland; ^3^Department of Analytical Chemistry, Chemical Faculty, Gdańsk University of Technology, Gdańsk, Poland

**Keywords:** endocrine disruptors, chemicals, bisphenol, hypothyroidism, thyroid function, thyroid volume, pregnancy, reproduction

## Abstract

Bisphenols (BPs) are commonly known plastifiers that are widely used in industry. The knowledge about the impact of BPs on thyroid function is scarce. Proper thyroid functioning is especially important for women of reproductive age, as hypothyroidism affects fertility, pregnancy outcomes and the offspring. There are no studies analyzing the influence of BPs on thyroid function and volume in non-pregnant young women. The aim of this cross-sectional study was to evaluate the relationship between bisphenol A and its 10 analogs (BPS, BPC, BPE, BPF, BPG, BPM, BPP, BPZ, BPFL, and BPBP) on thyroid function and volume in women of reproductive age. Inclusion criteria were: female sex, age 18–40 years. Exclusion criteria were history of any thyroid disease, pharmacotherapy influencing thyroid function, pregnancy or puerperium, and diagnosis of autoimmune thyroid disease during this study. Venous blood was drawn for measurement of thyrotropin (TSH), free thyroxine, thyroid peroxidase antibodies, thyroglobulin antibodies, BPs. Urine samples were analyzed for: ioduria and BPs. Ultrasound examination of thyroid gland was performed. One hundred eighty participants were included into the study. A negative correlation was found between urine BPC and the thyroid volume (R = −0.258; p = 0.0005). Patients with detected urine BPC presented smaller thyroid glands than those with not-detected urine BPC (p = 0.0008). A positive correlation was found between TSH and urine BPC (R = 0.228; p = 0.002). Patients with detected urine BPC presented higher concentrations of TSH versus those with not-detected urine BPC (p = 0.003). There were no relationships between any of serum BPs as well as the other urine BPs and thyroid function and its volume. The only BP that demonstrated the relationship between thyroid function and its volume was BPC, probably because of its chemical structure that most resembles thyroxine. Exposure to this BP may result in the development of hypothyroidism that could have a negative impact on pregnancy and the offspring.

## Introduction

Bisphenol A (BPA; 4,4’-isopropylidenediphenol) was first developed in 1891 as a synthetic estrogen and in the 1930s has been shown to stimulate the female reproductive system in rats. Because of the ability to interfere with the action of hormones, BPA belongs to the class of endocrine disrupting chemicals (EDC) (1–3). Presently, estrogenic properties of BPA have been forgotten and this chemical is commonly used in the production of polycarbonate plastics, including clear plastic bottles, plastic toys, baby pacifiers, teethers as well as epoxy resins, inner linings of food and beverage containers, dental sealants and thermal receipt paper (4–9). Because of research findings that suggest adverse effects on human health after exposure to BPA, international governments have denied the usage of this chemical in products dedicated for food storage and for children, especially for newborns. For this reason, numberous structural analogs of BPA have been used in growing quantities in the plastic industry and have been thought to be potentially safe alternatives of BPA. However, the knowledge of their biological and environmental impact is still limited. This is especially worrisome given multiple routes of human exposure, namely oral, transdermal, and inhalatory (10).

The most studied bisphenol, BPA, shows numerous adverse development effects including estrogen dependent neoplasms (11–13); metabolic disorders, such as obesity, diabetes, insulin resistance (14); polycystic ovary syndrome (15); and neurobehavioral disorders (16). There are also several studies that documented the harmful effects of BPA on the thyroid function (17–19).

Proper thyroid functioning is crucial for the maintenance of homeostasis in human organisms. For women of reproductive age, attaining euthyroidism is critically important since hypothyroidism affects fertility, pregnancy outcomes, and the health of the offspring. Pregnant women with overt or subclinical hypothyroidism have a higher risk of preeclampsia, premature delivery, premature abruption of placenta and miscarriage (20–22). Maternal hypothyroidism can also lead to the retardation of neural development of the fetus, low birth weight and intrauterine growth retardation of the offspring (20–22). According to American Thyroid Association guidelines from 2011 and European Thyroid Association guidelines from 2014 the upper reference limit for thyrotropin (TSH) in pregnancy was defined as <2.5 mU/L in the first trimester and <3.0–3.5 mU/l in the second and third trimesters (23, 24).

Most studies evaluating the effects of different bisphenols (BPs) on thyroid function were performed *in vitro* (19, 25, 26) or on animal models (mainly on rodents and zebrafish) (18, 27–29), while human studies have examined pregnant women and children (17, 30–32). Moreover, data on the impact of BPA analogs on the human thyroid function is limited. Several studies indicate that BPs may act as agonists of the thyroid hormone receptors (TR) (19), while others indicate that BPA also acts as an antagonist (18, 27). However, no studies have analyzed the influence of BPA and its analogs on the thyroid function and volume in non-pregnant women of reproductive age.

Therefore, the aim of this cross-sectional study was to evaluate the potential relationship between BPA and its 10 analogs on the thyroid function and volume in women of reproductive age living in Warsaw, Poland. These 10 analogs include bisphenol S (BPS), bisphenol C (BPC), bisphenol E (BPE), bisphenol F (BPF), bisphenol G (BPG), bisphenol M (BPM), bisphenol P (BPP), bisphenol Z (BPZ), bisphenol FL (BPFL), and bisphenol BP (BPBP).

## Materials and Methods

### Patients

This cross-sectional, clinical study was conducted in the Department of Internal Medicine and Endocrinology, Medical University of Warsaw in the period between October 2017 and May 2019. The study protocol conforms to the ethical guidelines of the 1975 Declaration of Helsinki as reflected in *a priori* approval by the institution’s human research committee. The study was approved by the Committee on Bioethics at the Medical University of Warsaw (nr KB/104/2015). Written informed consent was obtained from all participants.

**Figure 1:**
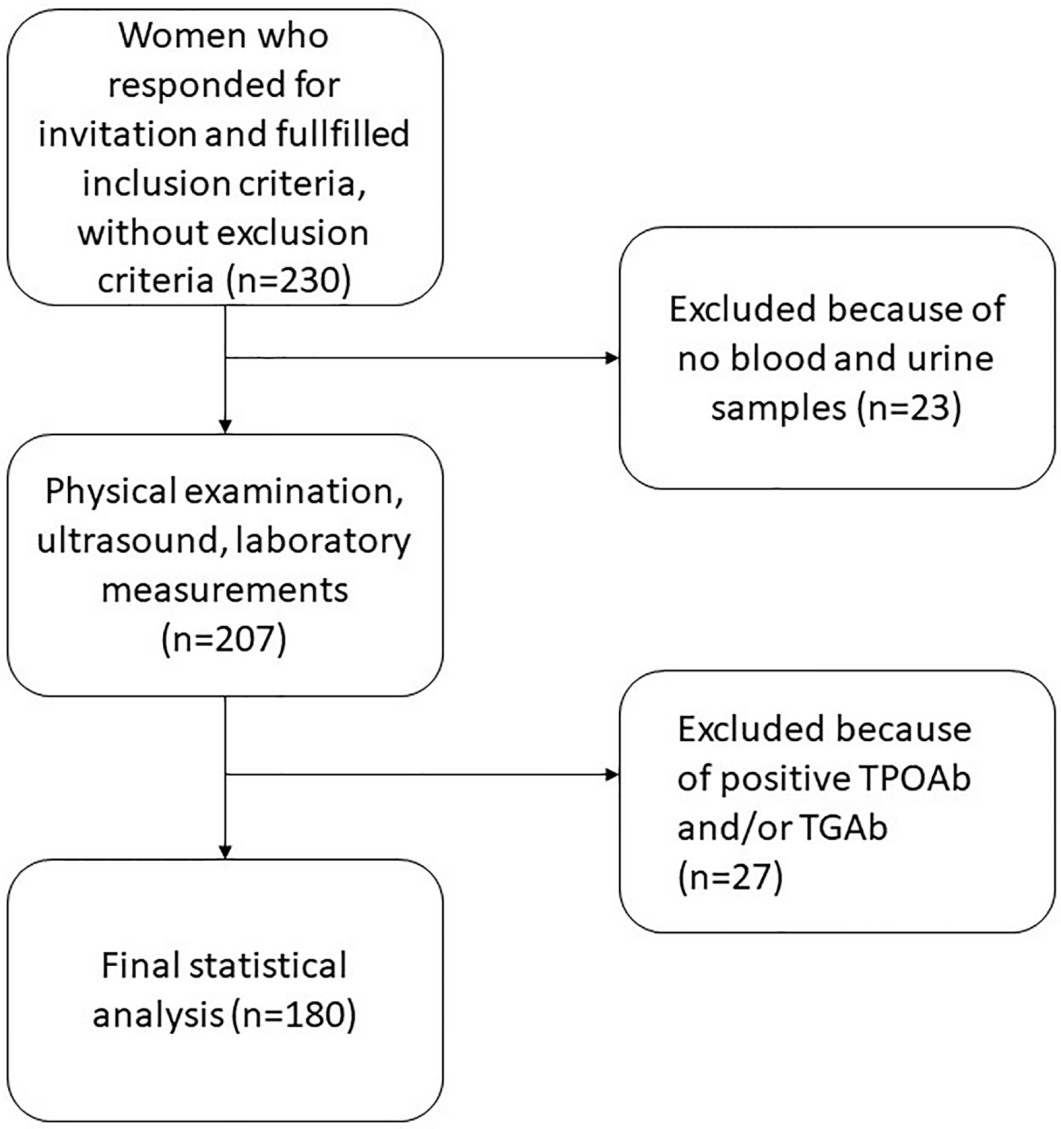
Flowchart diagram for the recruitment for the study. TPOAb, thyroid peroxidase antibodies; TGAb, thyroglobulin antibodies.

Inclusion criteria were female sex and age between 18 and 40 years. Exclusion criteria were as follows: (i) a documented history of no thyroid disease before the study (hypothyroidism, hyperthyroidism, thyroid nodules, and autoimmune thyroid disease); (ii) pharmacotherapy influencing the thyroid function (especially iodine, antithyroid drugs, levothyroxine, and selenium); (iii) pregnancy or puerperium; and (iv) diagnosis of autoimmune thyroid disease during this study. Finally, 180 women were eligible for the study. All were students of Medical University of Warsaw. All of them were research volunteers. Detailed characteristics of the studied subjects are contained in **Table 1**. Flowchart diagram for the recruitment for the study is presented in **Figure 1**.

**Table 1:** Basal characteristics of the study population (n = 180).

Characteristics	mean ± SD or n (%)
Age (years) BMI (kg/m^2^) Thyroid volume (ml) Laboratory measurements TSH (uIU/ml) (N: 0.27–4.2) fT4 (pmol/l) (N: 12–22) ioduria (ug/l) (N: 100–300) ioduria/g creatinine Presence of thyroid lesions in US Smoking current former never Pregnancy in the past	24 ± 3 21.2 ± 3.0 10.2 ± 3.2 2.1 ± 0.9 16.3 ± 2.2 122.0 ± 103.9 85.5 ± 103.3 58 (32) 20 (11) 27 (15) 133 (74) 9 (5)

BMI, body mass index; fT4, free thyroxine; SD, standard deviation; TSH, thyrotropin; US, ultrasonography.

### Thyroid Ultrasound Examination

Ultrasonography of the thyroid gland was performed by physician with a 5-year experience (JMB). All the ultrasound scans were performed using the Hitachi Avius Medical ultrasound technique system equipped with the 7.5–12 MHz high frequency linear array transducer. Thyroid ultrasound procedures were done on the basis of the Ultrasound Examination Standards of the Polish Ultrasound Society and American College of Radiology Guidelines (33, 34). The neck was scanned in sagittal, transverse and oblique sections to optimally visualize both lobes of the thyroid gland and the isthmus. All images were examined on real-time two-dimensional B-mode grayscale and Doppler imaging.

### Laboratory Measurements

All procedures were performed with precautions intended to minimize the risk of sample contamination with BPs. Venous blood was drawn from all study participants, in the morning, after an overnight fasting (>8 h) into 7-ml glass tubes. After 30 min of incubation in the room temperature the blood samples were centrifuged for 15 min (g-force 1,000xg). Next, serum samples were collected into 1.5-ml glass vials with polytetrafluoroethylene caps using disposable transfer (Pasteur) pipette made of low-density polyethylene. Serum samples were frozen and stored vertically at −70°C for further analyses. Additionally, each study participant was asked to bring 100 ml of midstream morning urine sample for the evaluation of urinary iodine, creatinine and BPs. Urine samples were also collected into glass jars, then frozen and stored vertically at −70°C for further analyses.

TSH, free thyroxine (fT4), thyroid peroxidase antibodies and thyroglobulin antibodies were measured in serum samples using an electrochemiluminescence immunoassay (Roche Diagnostics, Mannheim, Germany) on Cobas e411 Analyzer (Hitachi, Tokyo, Japan) in the Scientific Laboratory at the Department of Internal Medicine and Endocrinology, Medical University of Warsaw. Serum thyroid peroxidase antibodies and thyroglobulin antibodies above >34 and >115 IU/ml, respectively, were considered as “positive”.

Urine iodine concentrations were measured by the catalytic arsenium-cerium method based on Sandell-Kolthoff reaction (35). Urinary creatinine was assessed by colorimetric Jaffe method (36).

**Figure 2:**
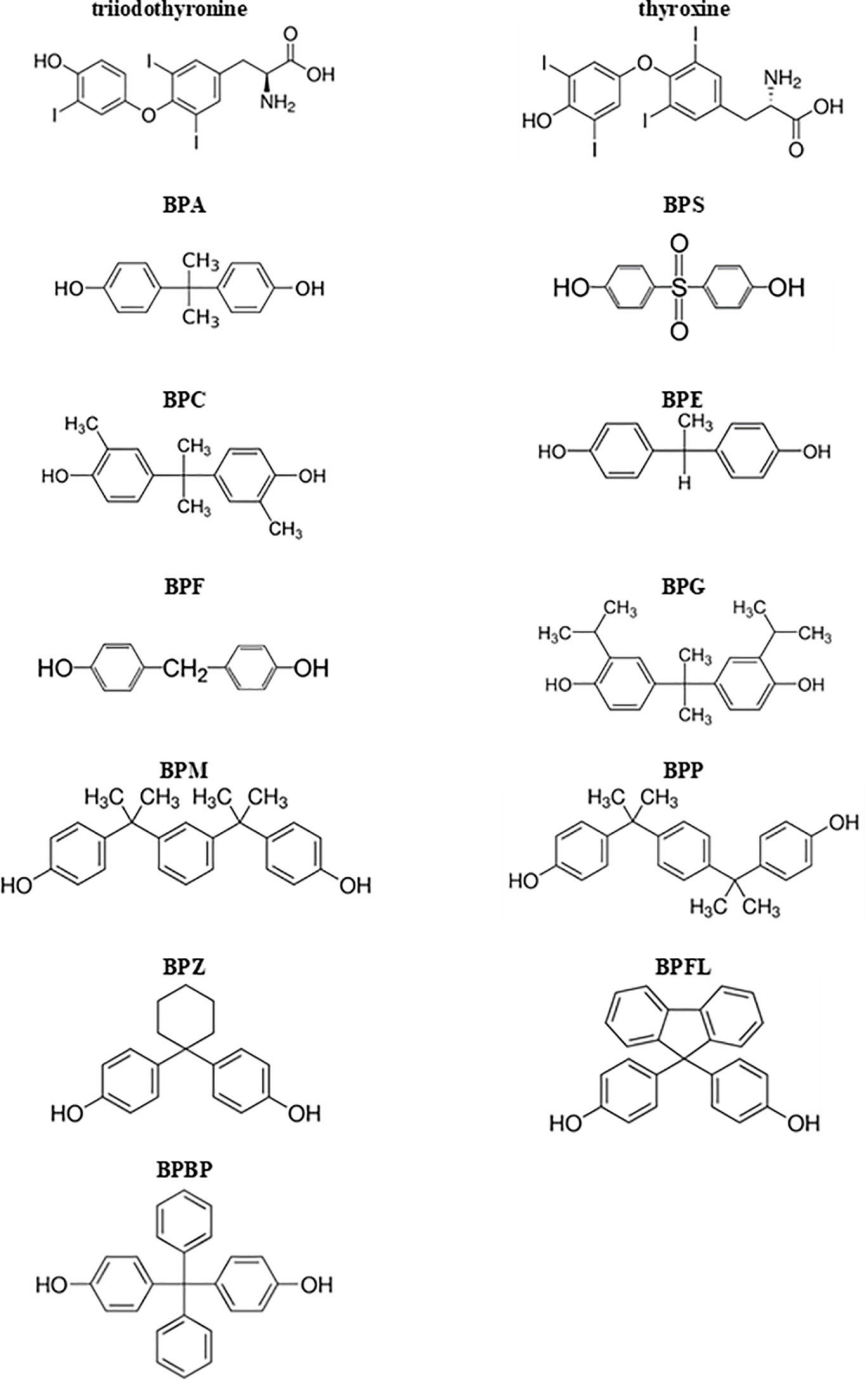
Chemical structures of bisphenols examined in this study and of thyroid hormones (triiodothyronine and thyroxine). BPA, bisphenol A; BPS, bisphenol S; BPC, bisphenol C; BPE, bisphenol E; BPF, bisphenol F; BPG, bisphenol G; BPM, bisphenol M; BPP, bisphenol P; BPZ, bisphenol Z; BPFL, bisphenol FL; BPBP, bisphenol BP.

Serum and urine concentrations of BPA and its 10 analogs (BPS, BPC, BPE, BPF, BPG, BPM, BPP, BPZ, BPFL, and BPBP) were measured using high performance liquid chromatography method with tandem mass spectrometry (HPLC-MS/MS) on the Shimadzu triple quadrupole LC-MS/MS system (LCMS-8060; Shimadzu. Japan) equipped with an electrospray ionization source working in the negative multiple reaction mode, in the Department of Analytical Chemistry, Chemical Faculty, Gdańsk University of Technology. Detailed description of the whole method has been already published elsewhere (37). The limit of detection (LOD) and limit of quantification (LOQ) for BPA and its 10 analogs are presented in **Table S1**. The term “detected” means that the concentration of the measured BP was above the LOD presented in **Table S1**. The term, “not-detected” means that the concentration of the measured BP was below the LOD presented in **Table S1**. BPs concentration values between the LOD and LOQ were extrapolated from the calibration curve. The term “detection rate (DR%)” means the percentage of women with the measurable BPs concentration above the LOD. Chemical structures of BPs tested in our study are shown in **Figure 2**.

### Statistical Approach and Analyses

All statistical analyses were performed with the usage of the computer program STATISTICA ver.13.0 for Windows. Distributions of the variables were evaluated with the Shapiro-Wilk test. Continuous variables are presented as arithmetic mean ± standard deviation (SD). Categorical data are presented as numbers and percentage values (%).

The analysis of the results consisted of the following steps: First, correlations were performed between concentrations of selected BPs and thyroid volume, TSH and fT4 using the Spearman correlation test. Second, comparisons of thyroid volume, TSH and fT4 were performed in two independent groups of participants (with detected versus not-detected concentrations of studied BPs) using the Mann-Whitney U test.

Because of the multiple comparisons, the Bonferroni correction was used and all results with p value < 0.0033 were considered statistically significant.

### Data Sets Availability

Database analyzed in this study is available for interested researchers in **Table S2**.

## Results

DR as well as mean serum and urine concentrations for each of the studied BP are presented in **Table S1**.

### Relationship Between Thyroid Volume and BPs Concentrations (Respectively in Serum and Urine)

All correlations between thyroid volume and concentrations of BPs, respectively in serum and urine, are given in **Table 2**. There were no statistically significant correlations between thyroid volume and serum BPs. There was a significant negative correlation between thyroid volume and urine concentration of BPC (R = −0.258; p = 0.0005). There were no correlations between thyroid volume and urine concentration of the other BPs.

**Table 2:** Correlations between concentrations of selected bisphenols in serum and urine and thyroid volume.

	R	p
Serum		
BPA BPS BPC BPE BPF BPG BPM BPP BPZ BPFL BPBP	−0.009 −0.027 0.049 −0.059 0.038 −0.161 −0.088 0.089 −0.049 0.013 −0.176	0.902 0.729 0.519 0.438 0.623 0.034 0.246 0.250 0.521 0.863 0.020
Urine		
BPA BPS BPC BPE BPF BPG BPM BPP BPZ BPFL BPBP	−0.069 −0.069 −0.258 0.005 −0.005 −0.040 −0.068 −0.037 0.029 −0.072 −0.009	0.365 0.360 0.0005* 0.945 0.943 0.598 0.368 0.624 0.698 0.344 0.905

BPA, bisphenol A; BPS, bisphenol S; BPC, bisphenol C; BPE, bisphenol E; BPF, bisphenol F; BPG, bisphenol G; BPM, bisphenol M; BPP, bisphenol P; BPZ, bisphenol Z; BPFL, bisphenol FL; BPBP, bisphenol BP.

Results were calculated for 176 serum samples and for 177 urine samples.

Correlations were evaluated with Spearman correlation coefficient. After Bonferroni correction results were claimed statistically significant with p value < 0.0033 (*).

**Figure 3:**
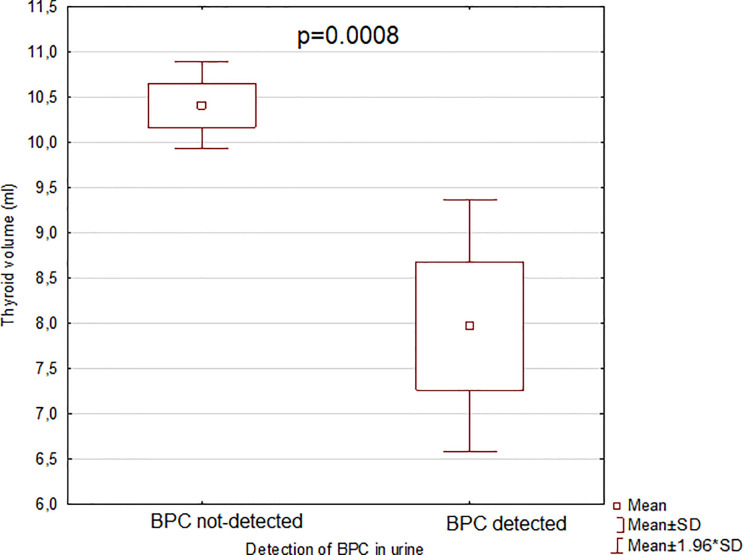
Comparison of thyroid volume according to the detection of bisphenol C (BPC) in urine (n = 177). BPC, bisphenol C; SD, standard deviation. The term “detected” means that the concentration of bisphenol C was above the limit of detection (LOD) presented in **Table S1**, see **Supplemental Material**. The term “not-detected” means that the concentration of bisphenol C was below the LOD. The comparison was performed with the Mann-Whitney U test. After Bonferroni correction results were claimed statistically significant with p < 0.0033.

All results from the comparison of thyroid volume according to the detection of the analyzed BPs (respectively in serum and urine) are presented in **Table 3**. No significant differences were found in thyroid volume between women with detected versus women with not-detected BPs in serum. Patients with detected BPC in urine presented significantly smaller thyroids than those with not-detected BPC in urine (p = 0.0008) (**Figure 3**). There were no significant differences in thyroid volumes of participants with detected versus women with not-detected BPs in urine.

**Table 3:** Comparison of thyroid volume (ml) according to the detection of selected bisphenols in serum and urine.

	BP detected	BP not-detected	p
Serum			
BPA BPS BPC BPE BPF BPG BPM BPP BPZ BPFL BPBP	10.1 ± 3.3 10.1 ± 2.8 10.5 ± 3.1 10.1 ± 3.1 10.1 ± 3.2 9.6 ± 2.9 10.0 ± 3.2 10.3 ± 3.0 10.1 ± 3.3 10.1 ± 2.8 9.5 ± 3.0	10.6 ± 3.0 10.3 ± 3.7 10.1 ± 3.2 10.3 ± 3.3 10.3 ± 3.2 10.5 ± 3.3 10.6 ± 3.1 10.1 ± 3.6 10.4 ± 3.1 10.4 ± 3.8 10.7 ± 3.3	0.291 0.969 0.465 0.644 0.778 0.097 0.135 0.558 0.487 0.963 0.022
Urine			
BPA BPS BPC BPE BPF BPG BPM BPP BPZ BPFL BPBP	10.2 ± 3.1 9.9 ± 3.1 8.0 ± 3.1 10.1 ± 3.0 10.2 ± 3.2 10.0 ± 3.5 10.0 ± 3.1 10.2 ± 3.3 10.1 ± 3.3 9.8 ± 3.0 10.0 ± 3.2	10.0 ± 3.3 10.4 ± 3.2 10.4 ± 3.1 10.2 ± 3.2 10.0 ± 3.0 10.2 ± 3.1 10.3 ± 3.2 10.1 ± 3.0 10.1 ± 3.0 10.3 ± 3.3 10.2 ± 3.2	0.680 0.200 0.0008* 0.981 0.876 0.480 0.503 0.855 0.975 0.314 0.922

BP, bisphenol; BPA, bisphenol A; BPS, bisphenol S; BPC, bisphenol C; BPE, bisphenol E; BPF, bisphenol F; BPG, bisphenol G; BPM, bisphenol M; BPP, bisphenol P; BPZ, bisphenol Z; BPFL, bisphenol FL; BPBP, bisphenol BP.

The term “detected” means that the concentration of selected bisphenol was above the limit of detection (LOD) presented in **Table S1**, see **Supplemental Material**. The term “not-detected” means that the concentration of selected bisphenol was below the LOD.

Results were calculated for 176 serum samples and for 177 urine samples.

Comparisons were performed with the Mann-Whitney U test. After Bonferroni correction results were claimed statistically significant with p value < 0.0033 (*).

### Relationship Between Thyroid Function and BPs Concentrations (Respectively in Serum and Urine)

All correlations between TSH and fT4 with BPs concentrations in serum and urine are presented in **Table 4**. There were no significant correlations between TSH as well as fT4 and serum concentration of BPs. There was a significant positive correlation between TSH and urine BPC concentrations (R = 0.228; p = 0.002). No significant correlations were observed between TSH and fT4 with urine concentrations of the other BPs.

**Table 4:** Correlations of bisphenols (BPs) concentrations in serum and urine with thyrotropin (TSH) and free thyroxine (fT4).

	BPs vs. TSH		BPs vs. fT4	
	R	p	R	p
Serum				
BPA BPS BPC BPE BPF BPG BPM BPP BPZ BPFL BPBP	0.044 0.007 −0.088 −0.005 0.055 0.066 0.013 −0.066 −0.081 0.012 0.079	0.562 0.932 0.245 0.953 0.468 0.387 0.867 0.383 0.287 0.872 0.295	−0.151 0.049 −0.021 0.014 −0.113 0.061 −0.002 −0.033 −0.026 0.044 −0.006	0.045 0.517 0.778 0.853 0.137 0.421 0.978 0.667 0.731 0.559 0.933
Urine				
BPA BPS BPC BPE BPF BPG BPM BPP BPZ BPFL BPBP	0.069 −0.210 0.228 0.026 0.039 −0.012 0.085 −0.066 −0.069 −0.056 0.014	0.360 0.005 0.002* 0.728 0.604 0.869 0.262 0.382 0.359 0.458 0.852	0.031 −0.010 −0.021 0.076 0.064 0.147 0.016 -0.095 0.046 0.066 0.069	0.679 0.890 0.781 0.317 0.399 0.050 0.832 0.206 0.541 0.383 0.360

BPs, bisphenols; BPA, bisphenol A; BPS, bisphenol S; BPC, bisphenol C; BPE, bisphenol E; BPF, bisphenol F; BPG, bisphenol G; BPM, bisphenol M; BPP, bisphenol P; BPZ, bisphenol Z; BPFL, bisphenol FL; BPBP, bisphenol BP; fT4, free thyroxine; TSH, thyrotropin.

Results were calculated for 176 serum samples and for 177 urine samples.

Correlations were evaluated with Spearman correlation coefficient. After Bonferroni correction results were claimed statistically significant with p value < 0.0033 (*).

**Figure 4:**
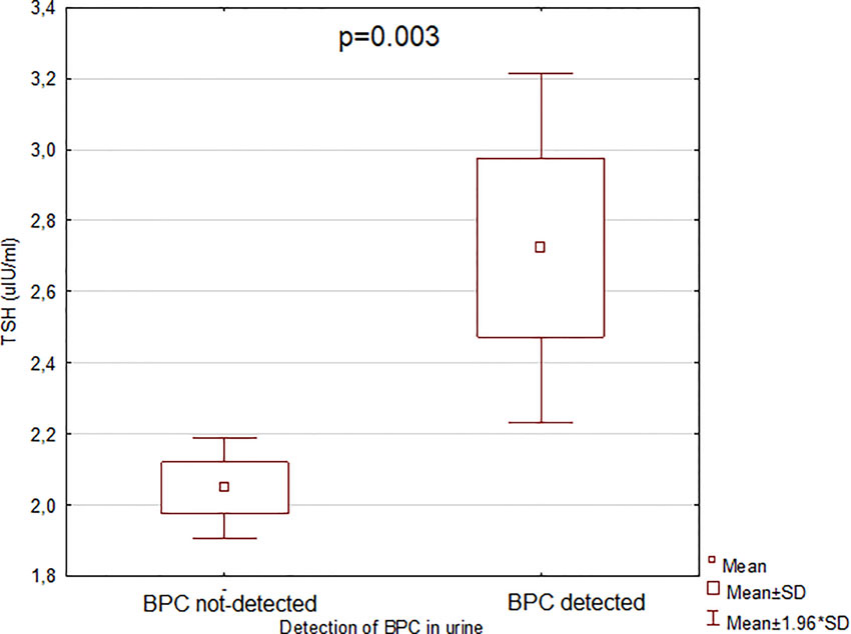
Relationship between thyrotropin (TSH) and bisphenol C (BPC). BPC, bisphenol C; TSH, thyrotropin. The term “detected” means that the concentration of bisphenol C was above the limit of detection (LOD) presented in **Table S1**, see **Supplemental Material**. The term “not-detected” means that the concentration of bisphenol C was below the LOD. The comparison was performed with the Mann-Whitney U test. After Bonferroni correction results were claimed statistically significant with p < 0.0033.

The results from the comparison of TSH and fT4 levels according to the detection of selected BPs in serum and urine are shown in **Table 5**. There were no statistically significant differences in TSH and fT4 levels between the groups of women with detected vs not-detected BPs in serum. Considering urine concentrations of BPs, only women with BPC detected in urine presented higher TSH levels versus those with not-detected urine BPC (p = 0.0033) (**Figure 4**). The comparison of fT4 levels between women with detectable vs non-detectable BPs in urine did not reveal any significant differences.

**Table 5:** Comparison of thyrotropin (TSH) level (mean ± SD; µIU/ml) and free thyroxine (fT4) level (mean ± SD; pmol/l) according to the detection of selected bisphenols in serum and urine.

	TSH (mean ± SD; µIU/ml)			fT4 (mean ± SD; pmol/l)		
	BP detected	BP not-detected	p	BP detected	BP not-detected	p
Serum						
BPA BPS BPC BPE BPF BPG BPM BPP BPZ BPFL BPBP	2.12 ± 0.96 2.07 ± 0.89 1.94 ± 0.78 2.13 ± 1.04 2.15 ± 0.99 2.26 ± 1.10 2.13 ± 1.00 2.11 ± 1.01 2.13 ± 0.99 2.09 ± 0.94 2.18 ± 0.99	2.00 ± 0.88 2.10 ± 1.01 2.15 ± 0.99 2.08 ± 0.89 2.04 ± 0.89 2.04 ± 0.87 2.04 ± 0.82 2.09 ± 0.83 2.07 ± 0.90 2.12 ± 0.97 2.04 ± 0.91	0.613 0.767 0.279 0.975 0.564 0.380 0.882 0.586 0.962 0.869 0.446	16.2 ± 2.1 16.3 ± 2.1 16.3 ± 2.6 16.3 ± 2.3 16.1 ± 2.1 16.5 ± 2.3 16.2 ± 2.1 16.3 ± 2.1 16.2 ± 2.3 16.4 ± 2.1 16.3 ± 2.4	16.6 ± 2.3 16.2 ± 2.2 16.3 ± 2.0 16.2 ± 2.1 16.5 ± 2.2 16.2 ± 2.1 16.5 ± 2.3 16.2 ± 2.3 16.3 ± 2.0 16.1 ± 2.3 16.2 ± 2.0	0.452 0.646 0.237 0.929 0.340 0.467 0.548 0.996 0.852 0.336 0.866
Urine						
BPA BPS BPC BPE BPF BPG BPM BPP BPZ BPFL BPBP	2.17 ± 0.97 1.94 ± 0.89 2.72 ± 1.09 2.14 ± 0.95 2.11 ± 0.99 2.02 ± 0.75 2.19 ± 1.00 2.03 ± 0.88 2.05 ± 1.01 2.08 ± 0.92 2.23 ± 1.11	1.92 ± 0.84 2.29 ± 0.97 2.05 ± 0.90 2.11 ± 0.95 2.17 ± 0.82 2.14 ± 0.98 2.02 ± 0.86 2.24 ± 1.02 2.19 ± 0.88 2.15 ± 0.96 2.07 ± 0.86	0.197 0.009 0.003* 0.713 0.435 0.902 0.442 0.188 0.138 0.750 0.745	16.4 ± 2.1 16.3 ± 2.0 16.1 ± 1.4 16.5 ± 2.1 16.3 ± 2.1 17.0 ± 2.0 16.2 ± 2.1 16.2 ± 2.2 16.3 ± 2.1 16.4 ± 2.1 16.4 ± 2.1	15.6 ± 2.0 16.2 ± 2.2 16.3 ± 2.2 16.1 ± 2.1 16.2 ± 2.2 16.1 ± 2.1 16.3 ± 2.2 16.4 ± 2.1 16.2 ± 2.1 16.1 ± 2.1 16.2 ± 2.1	0.041 0.768 0.839 0.356 0.721 0.042 0.927 0.479 0.630 0.167 0.402

BP, bisphenol; BPA, bisphenol A; BPS, bisphenol S; BPC, bisphenol C; BPE, bisphenol E; BPF, bisphenol F; BPG, bisphenol G; BPM, bisphenol M; BPP, bisphenol P; BPZ, bisphenol Z; BPFL, bisphenol FL; BPBP, bisphenol BP; fT4, free thyroxine; TSH, thyrotropin.

The term “detected” means that the concentration of selected bisphenol was above the limit of detection (LOD) presented in **Table S1**, see **Supplemental Material**. The term “not-detected” means that the concentration of selected bisphenol was below the LOD.

Results were calculated for 176 serum samples and for 177 urine samples.

Comparisons were performed with the Mann-Whitney U test. After Bonferroni correction results were claimed statistically significant with p value < 0.0033 (*).

## Discussion

BPA analogs are widely used in many applications replacing BPA, but without proper understanding of their harmful potential. Human and wildlife observations point toward subtle, but significant effects of low-dose BPs exposure on the thyroid function. However, mechanistic and toxicological aspects as well as epidemiological design are necessary for the objective assessment of the impact of BPs on humans. Hence, this demands a great challenge for scientists who have to examine the possible toxicity of a selected molecule.

DR of BPA in studies was mainly at over 90% (30, 38, 39). In the present study, the DR of BPA in serum and urine samples was slightly lower, at about 80% of samples. Data on the DRs of BPA analogs in human studies are poor. Notably, there are no data on the DR of BPC in humans.

The difference in the level of DRs between studies is probably caused by living environment of the participants. Moreover, different methods of BPs detection were used (HPLC, HPLS-MS/MS, LC-MS, GC-MS). Also, the relatively high LOD for the method used could be another reason for lower DR of BPs in the current study. Furthermore, it is worth to emphasize the fact that in the studies there were examined conjugated, unconjugated or total BPs (mainly BPA). Also the stability of phenolic compounds differs according to the type of sample (blood, urine, tissues) and the method of samples storage (10).

Our findings examining the relationships between BPs and thyroid volume showed only urine concentration of BPC was correlated with thyroid volume. The correlation was negative, signifying those with higher BPC concentrations had smaller thyroid volumes. A possible reason for the difference between our results in urine and serum could be variations in the stability of phenolic compounds (BPs) in fluids (10). To our knowledge there are no studies on the relationship between BPC and thyroid volume, neither in human, nor in animals or *in vitro*. In the study on 718 children of school-age living in China, Wang et al. (30) reported the negative correlation between urinary BPA and thyroid volume.

BPA has been shown to bind to the TR and therefore, affect the thyroid hormone signaling *in vitro*. Moriyama et al. (18) focused their attention on the resemblance of the chemical structures between BPA and thyroid hormone triiodothyronine (T3), where two benzene cores are linked by carbon (BPA) or oxygen (T3). The authors showed that BPA inhibits TR-mediated transcription by acting as an antagonist, suppressing transcriptional activity that is stimulated by T3 in a dose-dependent manner, but only in the presence of physiological concentrations of T3. Zoeller et al. (27) in an *in vivo* study on rats during pregnancy and lactation reported that dietary exposure to BPA led to an increase in serum T4 in pups, but did not alter serum TSH. These results are in contradiction to ours, where urine BPC concentration correlated positively with serum TSH, but not fT4 levels (in the current study T4 has not been measured). To the best of our knowledge no studies on human or animals have considered the influence of exposure to BPC on thyroid function. The change of thyroid volume together with the increase of TSH with unaltered fT4 seems to happen only at subclinical level. We suggest that thyroids exposed to BPC secrete less amounts of thyroid hormones, but without statistical significance in fT4 levels. As a consequence, the level of TSH increases. In case of pregnancy overt hypothyroidism may be revealed that could contribute to an increased risk of neurodevelopmental disease, learning difficulties, language delay and IQ loss in offspring (40–42).

Kitamura et al. (19) performed a comparative study of the endocrine-disrupting activity of BPA and 19 related compounds in rats. Results from this *in vitro* study indicated significant thyroid hormonal activity toward rat pituitary cell line GH3 of the following selected BPA derivatives: tetrabromobisphenol A (TBBPA), tetrachlorobisphenol A (TCBPA), tetramethylbisphenol A (TMBPA), and 3,3’-dimethylbisphenol A. However, BPA and other analogs (such as BPF, BPS, and BPAF) did not show such action. These authors suggest that the 4-hydroxyl groups of the A phenyl ring and the B phenyl ring of BPA analogs are essential for these hormonal activities. Moreover, substituents at the 3,5-positions of the phenyl rings and the bridging alkyl moiety significantly modifies the thyroid hormonal activity. This could be the reason why in our study the only one BP that exhibited the relationship with thyroid functioning was BPC. The study presents strong relationship between thyroid and BPC, while the rest of the analyzed BPs did not show statistically significant trend for such relationships. Therefore, we suggest that the chemical structure of BPC most resembles T4 in that substituents of phenyl rings are as iodine ions in T4.

Hence, thyroid hormone disruption by BPs may not be solely explained by the interaction with the TR. Reports in the literature suggest other ways that BPs dysregulate thyroid hormone homeostasis at the transcriptional level. Lee et al. (26) in the *in vitro* study on the rat pituitary cell line (GH3) and the thyroid follicular cell line (FRTL-5) clearly indicated the down-regulation effect on *tshβ*, *trα*, *trβ*, *dio1*, and *dio2* genes in GH3 cells caused by BPA and its selected derivatives. The analogs, such as BPF, BPM, and BPZ, presented even stronger potency compared to BPA. On the other hand, in FRTL-5 cells after exposure to BPA, BPAF, BPAP, BPM, or BPS, at least one gene responsible for either thyroid hormone synthesis (*nis*, *tg*, or *tpo*) or their transcription factors (*pax8* and *nkx2.1*) exhibited over 1.5-fold up-regulation. In contrast, BPC, BPF, BPP, and BPZ did not lead to transcriptional changes of >1.5-fold. The conclusion is that exposure to BPs could up-regulate or down-regulate gene transcription that depends on the cell line and the type of the BP.

An advantage of the present study is that it was conducted in the homogenous group of female participants at similar age. All were inhabitants of the same Mazovian district, thus eliminating the study bias reflecting the influence of the environmental factors. On the other hand, the small sample size and cross-sectional design are the limitations of this study. It would be interesting to examine women after follow-up as well as to conduct studies in other populations, such as pregnant women, children and men. Also, the study considering mixtures of thyroid EDCs and their synergies would be of a great value, as these are what we are most likely exposed to.

In conclusion, to the best of our knowledge our study is the first to evaluate the potential effects of BPA and its 10 analogs on the thyroid function and volume in women of reproductive age. This cross-sectional study presents that BPC impacts on the thyroid function and volume that might have negative effects on fertility and pregnancy outcomes in young women (43, 44). It is probably a result of resemblance in the chemical structure of BPC and T4. There is a strong need to conduct further studies in large human cohorts to estimate the relationship between BPA analogs and thyroid functioning and its morphology. Therefore, it is advised for the government to establish regulations for allowed thresholds and decide the authorization for common use of new chemicals.

## Data Availability Statement

The database analyzed in this study is available for interested researchers in **Table S2**, see **Supplemental Material**.

## Ethics Statement

The studies involving human participants were reviewed and approved by Committee on Bioethics, Medical University of Warsaw, Warsaw, Poland. The patients/participants provided their written informed consent to participate in this study.

## Author Contributions

Conceptualization: JM-B, PM, and TB. Methodology: JM-B, PM, KM-C, DR, and AW. Data collecting: JM-B and KM-C. Statistical analysis: JM-B. Writing—original draft preparation: JM-B and PM. Writing—review and editing: PM, TB, DR, AW, and KM-C. All authors contributed to the article and approved the submitted version.

## Conflict of Interest

The authors declare that the research was conducted in the absence of any commercial or financial relationships that could be construed as a potential conflict of interest.

## Abbreviations

BPs, bisphenols; BPA, bisphenol A; BPBP, bisphenol BP; BPC, bisphenol C; BPE, bisphenol E; BPF, bisphenol F; BPFL, bisphenol FL; BPG, bisphenol G; BPM, bisphenol M; BPP, bisphenol P; BPS, bisphenol S; BPZ, bisphenol Z; DR%, detection rate; EDC, endocrine disrupting chemicals; FRTL-5, a type of thyroid follicular cell line; fT4, free thyroxine; GH3, a type of rat pituitary cell line; LOD, limit of detection; LOQ, limit of quantification; SD, standard deviation; T3, triiodothyronine; TBBPA, tetrabromobisphenol A; TCBPA, tetrachlorobisphenol A; TMBPA, tetramethylobisphenol A; TR, thyroid hormone receptors; TSH, thyrotropin.
