# Supplementary material for: Exposure to Bisphenol A Analogs and the Thyroid Function and Volume in Women of Reproductive Age—Cross-Sectional Study
Source: Front Endocrinol (Lausanne). 2021 Jan 19;11:587252. doi: 10.3389/fendo.2020.587252 (PMC7851079; doi:10.3389/fendo.2020.587252)
Supplement: Supplementary file 1 [file Table_1.pdf]

Table S1. Serum and urine concentrations (ng/ml) of selected bisphenols.

	LOD	LOQ	DR%	mean	SD
**Serum**					
BPA	0.009	0.028	79	0.133	0.280
BPS	0.022	0.067	57	0.030	0.061
BPC	0.021	0.061	22	0.009	0.020
BPE	0.011	0.026	39	0.014	0.037
BPF	0.012	0.037	56	0.035	0.077
BPG	0.008	0.024	30	0.009	0.028
BPM	0.018	0.054	67	0.042	0.064
BPP	0.019	0.056	63	0.042	0.064
BPZ	0.017	0.051	56	0.049	0.089
BPFL	0.014	0.041	60	0.040	0.080
BPBP	0.012	0.120	43	0.026	0.038
**Urine**					
BPA	0.036	0.109	81	0.330	0.405
BPS	0.070	0.209	48	0.076	0.144
BPC	0.105	0.315	11	0.016	0.048
BPE	0.073	0.240	33	0.044	0.074
BPF	0.033	0.099	76	0.173	0.259
BPG	0.032	0.095	16	0.008	0.020
BPM	0.017	0.050	60	0.054	0.089
BPP	0.020	0.060	56	0.067	0.127
BPZ	0.035	0.106	49	0.057	0.081
BPFL	0.088	0.265	40	0.086	0.170
BPBP	0.068	0.205	32	0.049	0.113

BPA, bisphenol A; BPS, bisphenol S; BPC, bisphenol C; BPE, bisphenol E; BPF, bisphenol F; BPG, bisphenol G; BPM, bisphenol M; BPP, bisphenol P; BPZ, bisphenol Z; BPFL, bisphenol FL; BPBP, bisphenol BP; LOD, limit of detection; LOQ, limit of quantification; SD, standard deviation

Mean and SD are calculated for all patients independently of the detection in serum and urine.

DR%, detection rate – means the percentage of women with the concentration of selected bisphenols above the limit of detection (LOD).
